# Dynamic Changes of *Staphylococcus aureus* Susceptibility to Vancomycin, Teicoplanin, and Linezolid in a Central Teaching Hospital in Shanghai, China, 2008–2018

**DOI:** 10.3389/fmicb.2020.00908

**Published:** 2020-05-12

**Authors:** Ying Jian, Huiying Lv, Junlan Liu, Qian Huang, Yao Liu, Qian Liu, Min Li

**Affiliations:** Department of Laboratory Medicine, Renji Hospital, School of Medicine, Shanghai Jiao Tong University, Shanghai, China

**Keywords:** *Staphylococcus aureus*, minimum inhibitory concentration, vancomycin, teicoplanin, linezolid

## Abstract

Vancomycin, teicoplanin, and linezolid are the major treatment options for methicillin-resistant *Staphylococcus aureus* (MRSA). The phenomenon of progressive increase in the value of vancomycin minimum inhibitory concentration (MIC) for *S. aureus* (i.e., vancomycin MIC “creep”), has been reported; however, it is still a controversial concept because the results of research remain inconclusive. In this study, we conducted a retrospective epidemiologic investigation for more than 10 years to elucidate the dynamic changes of the MICs of vancomycin, teicoplanin, and linezolid in *S. aureus* in a central teaching hospital in Shanghai, China. A total of 2911 *S. aureus* isolates was recovered from 2008 to 2018, to which the MICs of three antimicrobials were tested by the E-test method and subsequently correlated with the characteristics of oxacillin susceptibility, clonotypes, and antimicrobial consumption during the study period. The proportion of MRSA dramatically decreased from 2008 to 2018 (from 84 to 49%, *p <* 0.001). Vancomycin MIC decline was identified both in MRSA and methicillin-sensitive *S. aureus* (MSSA) (both with *p <* 0.001), and both the dominating MRSA clone ST5 and pre-dominating MRSA clone ST239 displayed vancomycin MIC decline (*p <* 0.001, *p* = 0.040), while teicoplanin MIC decline was only identified in MRSA (*p* = 0.037). Linezolid MIC creep was identified in total *S. aureus* (*p* < 0.001), but linezolid in MRSA as well as teicoplanin and linezolid in MSSA displayed no statistically distinct trends of MIC creep or decline. Clinical consumption of linezolid increased significantly from 2012 to 2018 (*p* = 0.003), which correlated with vancomycin MIC decline in *S. aureus* (*p* = 0.005). The results of this study clearly demonstrate the dynamic changes of the MICs of these three primary antimicrobials in *S. aureus*, and suggest that changes in clinical antibiotic use may affect bacterial resistance.

## Introduction

*Staphylococcus aureus* can cause invasive or complicated infections, including bacteremia, pneumonia, osteoarticular infections, endocarditis, and skin and soft tissue infections (Lowy, 1998). MRSA was initially reported by Jevons in England in 1961, and then spread globally, causing extensive concern for its serious problems in both hospital and community (Enright et al., 2002). According to the latest work by China Antimicrobial Surveillance Network (CHINET), *S. aureus* ranked third in prevalence among all clinically isolated species and first among Gram-positive pathogens. While the prevalence of MRSA across China declined from 69% in 2008 to 35% in 2017, the isolation rate was much higher in Shanghai at about 49% in 2017 (Hu et al., 2018). Although its contribution to bacteremia infections varied throughout the world and fell generally in the past decades, MRSA still plays a crucial role in *S. aureus* infection, and it is reported that MRSA is associated with poorer clinical outcomes compared with methicillin-sensitive *S. aureus* (MSSA) (Gould, 2010; Tong et al., 2015). Vancomycin has long been the preferred primary treatment option since it was first introduced for the treatment of MRSA infection (Rodvold and McConeghy, 2014). However, vancomycin resistant *S. aureus* was first reported by Hiramatsu et al. (1997) in Japan which stimulated the development of new antibiotics to cure MRSA infection. Teicoplanin was first introduced to the market in 1989 in Italy, while linezolid was introduced in 2000 in the United States, both of which have been strikingly therapeutic in the current clinical treatment of MRSA infection despite such finite indications (Liu et al., 2011).

Recently, a phenomenon of progressive increase in the value of vancomycin minimum inhibitory concentration (MIC) for *S. aureus* was observed and reported in numerous studies as “MIC creep” (Steinkraus et al., 2007; Ho et al., 2010). However, MIC creep is still a controversial concept, as some groups drew different conclusions, or even opposite conclusions of MIC decline (Ruiz et al., 2016; Diaz et al., 2018). *S. aureus* isolates exhibiting high vancomycin MIC value relate to higher mortality and worse prognosis, for example, mortality associated with MRSA bacteremia was significantly higher with strains of high vancomycin MIC (>1 mg/L) (Soriano et al., 2008; Kalil et al., 2014). As the reported vancomycin therapy failure in patients with *S. aureus* infections with an MIC ≥ 4 mg/L, the Clinical and Laboratory Standards Institute (CLSI, 2006) halved vancomycin breakpoints from ≤4 to ≤2 mg/L (CLSI, 2006). Hence, in the present study, we retrospectively investigated *S. aureus* isolates from 2008 to 2018 in a tertiary care hospital, one of the biggest general hospitals in Shanghai, China. In order to find out if MIC creep taken place in *S. aureus* isolates in this hospital, and to figure out whether clone types or antimicrobial consumption would make a difference to dynamic changes of MICs, we investigated the dynamic changes of the MICs of three major clinical antimicrobials, vancomycin, teicoplanin, and linezolid, as well as their disparities between MRSA and MSSA, and correlated these changes with the clone types and antimicrobial consumption during the study period.

## Materials and Methods

### Ethics Statement

The bacteria from patient samples were approved by the ethics committee of Renji Hospital, School of Medicine, Shanghai Jiao Tong University, Shanghai, China. This project is a retrospective study. All of the *S. aureus* isolates were cultured and identified in routine microbiology laboratories. It did not involve the collection of patients’ clinical information, and did not interfere with patients’ clinical treatment. Patients were not involved in any way in the study, only molecular analysis of the bacteria was performed, thus, informed consent was not required for participation in this study.

### Bacterial Isolates

A total of 2911 sequential and non-repetitive *S. aureus* isolates were collected from a comprehensive teaching hospital in Shanghai, China from 2008 to 2018 (part of the isolates in 2010 and 2012 failed to revive). This is a centrally located large and particularly representative teaching hospital in Shanghai with 2000 beds and 10,000 admissions/day. In addition to routine microbiology/biochemical methods (such as Gram staining, catalase, and coagulase activity tests), MALDI-TOF-MS (Bruker Daltonics, Bremen, Germany) was used to further confirm the identities of *S. aureus* isolates. All isolates were stored at −80°C for later use.

### Antimicrobial Susceptibility Testing

The standard disk diffusion method was used to test oxacillin susceptibility (Oxiod, Basingstoke, United Kingdom) of all isolates. MICs of three major antimicrobials: vancomycin (Autobio, Zhengzhou, China), teicoplanin (Bio-Kont, Wenzhou, China), and linezolid (Autobio, Zhengzhou, China) were determined for each *S. aureus* isolate by E-test method, and the results were interpreted in accordance with CLSI guidelines. *S. aureus* ATCC29213 was used as a quality control strain.

### Molecular Typing Methods

Chromosomal DNA was extracted following culture on blood agar plates by a standard phenol-chloroform extraction procedure and used as a template for PCR reaction. Multi-locus sequence typing (MLST) was carried out according to the method described previously (Maiden et al., 1998). The DNA sequences of seven housekeeping genes were detected: carbamate kinase (*arcC*), shikimate dehydrogenase (*aroE*), glycerol kinase (*glp*), guanylate kinase (*gmk*), phosphate acetyltransferase (*pta*), triosephosphate isomerase (*tpi*), and acetyl coenzyme A acetyltransferase (*yqiL*). The sequence types (STs) were lastly determined by comparing the sequences of each gene to those of the known alleles deposited in the *S. aureus* MLST database^1^.

### Linezolid Resistance Mechanism Investigation

DNA was extracted from the linezolid resistant MRSA isolate grown on blood agar; then the presence of *cfr*, *optrA*, and *poxtA* genes was screened using previously described methods (Kehrenberg and Schwarz, 2006; Antonelli et al., 2018; Sassi et al., 2019); the mutations in 23S rRNA domain V were detected as well (Doern et al., 2016). The PCR primers of *cfr* (*cfr* forward: 5’-TGA AGT ATA AAG CAG GTT GGG AG, *cfr* reverse: 5′-ACC ATA TAA TTG ACC ACA AGC AGC), *optrA* (*optrA*-fw: 5′-ATG GTA ATA TGG TGT TGG AA, *optrA*-rev: 5′-TTG TAC AAA CTC TAC ACC AT), *poxtA* (*potxA*-fw: 5′-GGT CTG ACT GGC TTG TTT TGC T, *poxtA*-rev: 5′-ATA AGG TCG GTA TTG TCG GCG T), and 23S rRNA domain V (forward: 5′-AAC GAT TTG GGC ACT GTC TCA ACG, reverse: 5′-AAT TTC CTA CGC CCA CGA CGG ATA) were designed for targeted gene amplification. Products were visualized using agarose gel electrophoresis and subjected to Sanger sequencing, followed by comparing the generated sequence to the reference sequence from GenBank.

### Antimicrobials Usage Data

Data of vancomycin and linezolid daily defined doses (DDDs, an indicator of antimicrobials usage) were gathered and calculated by the Department of Pharmacy in this central teaching hospital.

### Statistical Analysis

The geometric mean MIC, MIC50, and MIC90 (MICs required to inhibit the growth of 50 and 90% of bacteria, respectively), and MIC range were evaluated mathematically. Statistical tests were performed with the GraphPad Prism software system. Categorical variables were compared using the chi-squared test or Fisher’s exact test. Dynamic change of MIC between years was carried out using non-parametric Spearman correlation test. Non-parametric method was also performed to determine the correlation between antimicrobials usage and MICs change. Differences between antimicrobials were analyzed using paired-sample geometric *t*-test. A *p*-value < 0.05 was considered statistically significant.

## Results

### Dynamic Changes of Defined MICs, 2008–2018

A total of 2911 sequential and non-repetitive *S. aureus* isolates were collected for this study (556 isolates in 2008, 277 isolates in 2010, 362 isolates in 2012, 487 isolates in 2015, 423 isolates in 2016, 409 isolates in 2017, and 397 isolates in 2018; besides, part of isolates in 2010 and 2012 failed to revive so the number we listed here was the actual number of isolates which were performed antibiotic susceptibility test). Geometric mean MIC, MIC50, MIC90, and MIC range of every single isolate to each antimicrobial are exhibited in Table 1. The geometric mean MIC of vancomycin, teicoplanin, and linezolid, respectively, was shifted from 1.18, 0.75, and 1.17 mg/L in 2008 to 0.92, 0.58, and 1.31 mg/L in 2018, suggesting that for *S. aureus* the MICs of glycopeptides declined, but those of oxazolidones increased. While taking MRSA or MSSA into consideration separately, we found that the variation was not so distinct as when MRSA and MSSA were assessed jointly.

**TABLE 1:** Defined MICs of vancomycin, teicoplanin, and linezolid in *S. aureus*, 2008–2018.

**Items**	**Antimicrobials**	Defined MIC*	**2008**	**2010**	**2012**	**2015**	**2016**	**2017**	**2018**
*S. aureus*	VAN	GEOmean	1.18	1.11	1.08	0.97	0.92	0.86	0.92
		MIC50	1	1	1	1	1	0.75	1
		MIC90	1.5	1.5	1.5	1.5	1.5	1	1.5
		MIC range	0.5-2	0.5-2	0.38-2	0.25-2	0.25-2	0.38-2	0.5-2
*S. aureus*	TCL	GEOmean	0.75	0.64	0.79	0.80	0.71	0.61	0.58
		MIC50	1	0.5	1	1	0.5	0.5	0.5
		MIC90	2	1	2	2	2	1	1
		MIC range	0.125-4	0.25-4	0.25-4	0.25-4	0.25-4	0.25-4	0.25-4
*S. aureus*	LZD	GEOmean	1.17	1.34	1.22	1.30	1.24	1.31	1.31
		MIC50	1	1.5	1.5	1.5	1.5	1.5	1.5
		MIC90	2	2	2	2	2	2	2
		MIC range	0.38-3	0.5-2	0.5-12	0.5-3	0.38-3	0.5-3	0.5-3
MRSA	VAN	GEOmean	1.19	1.14	1.16	1.01	0.97	0.87	0.95
		MIC50	1	1	1	1	1	0.75	1
		MIC90	1.5	1.5	1.5	1.5	1.5	1	1.5
		MIC range	0.5–2	0.75–2	0.38–2	0.25–2	0.38–2	0.5–2	0.5–1.5
MRSA	TCL	GEOmean	0.84	0.74	0.95	0.95	0.87	0.75	0.71
		MIC50	1	1	1	1	1	0.5	0.5
		MIC90	2	1	2	2	2	2	2
		MIC range	0.125–4	0.25–4	0.25–4	0.25–4	0.25–4	0.25–4	0.25–4
MRSA	LZD	GEOmean	1.16	1.31	1.16	1.24	1.19	1.22	1.26
		MIC50	1	1.5	1	1.5	1	1.5	1
		MIC90	2	2	2	2	2	1.5	2
		MIC range	0.38–3	0.5–2	0.5–12	0.5–3	0.38–3	0.5–3	0.75–3
MSSA	VAN	GEOmean	1.15	1.04	0.95	0.90	0.85	0.83	0.90
		MIC50	1	1	1.00	1.00	1	0.75	1
		MIC90	1.5	1.5	1.5	1.5	1	1	1
		MIC range	0.75–2	0.5–2	0.5–1.5	0.25–1.5	0.25–2	0.38–1.5	0.5–2
MSSA	TCL	GEOmean	0.43	0.45	0.56	0.58	0.51	0.49	0.48
		MIC50	0.5	0.5	0.5	0.50	0.5	0.5	0.5
		MIC90	1	1	1	1	1	1	1
		MIC range	0.125-2	0.25-2	0.25-2	0.25-2	0.25-2	0.25-1	0.25-2
MSSA	LZD	GEOmean	1.27	1.40	1.34	1.42	1.34	1.41	1.36
		MIC50	1.5	1.5	1.5	1.50	1.5	1.5	1.5
		MIC90	2	2	2	2	2	2	2
		MIC range	0.5–2	0.75–2	0.75–3	0.75–2	0.75–3	0.5–3	0.5–3

*A total of 2911 sequential and non-repetitive *S. aureus* isolates were collected for this study, including 1895 MRSA isolates and 1016 MSSA isolates. VAN: vancomycin; TCL: teicoplanin; LZD: linezolid. Defined MIC of geometric mean MIC (GEOmean), MIC50, MIC90, and MIC range were evaluated in this study; geometric mean MIC was the central number in a geometric distribution of MIC; MIC50, MIC90 were the middle and nine tenths value of orderly assembled MIC data array, respectively; MIC range was the literal meaning of range of MIC value in a targeted year.

We found that the MIC range of linezolid in 2012 being 0.5–12 mg/L, with the detection of an isolate with a linezolid MIC identified as 12 mg/L, which is so-called heterogeneous linezolid resistant *S. aureus* (hLRSA) in accordance with CLSI guidelines. The *cfr* gene, which was reported as a common mechanism generating linezolid resistance in gram positive cocci (Pantosti et al., 2007), was identified in this linezolid resistant isolate; while the *optrA* and *poxtA* genes were found to be absent and no mutations were detected in the 23S rRNA domain V of this isolate.

### MIC Decline for Vancomycin and Teicoplanin, and MIC Creep for Linezolid, Was Exhibited in *S. aureus* Isolates

**FIGURE 1:**
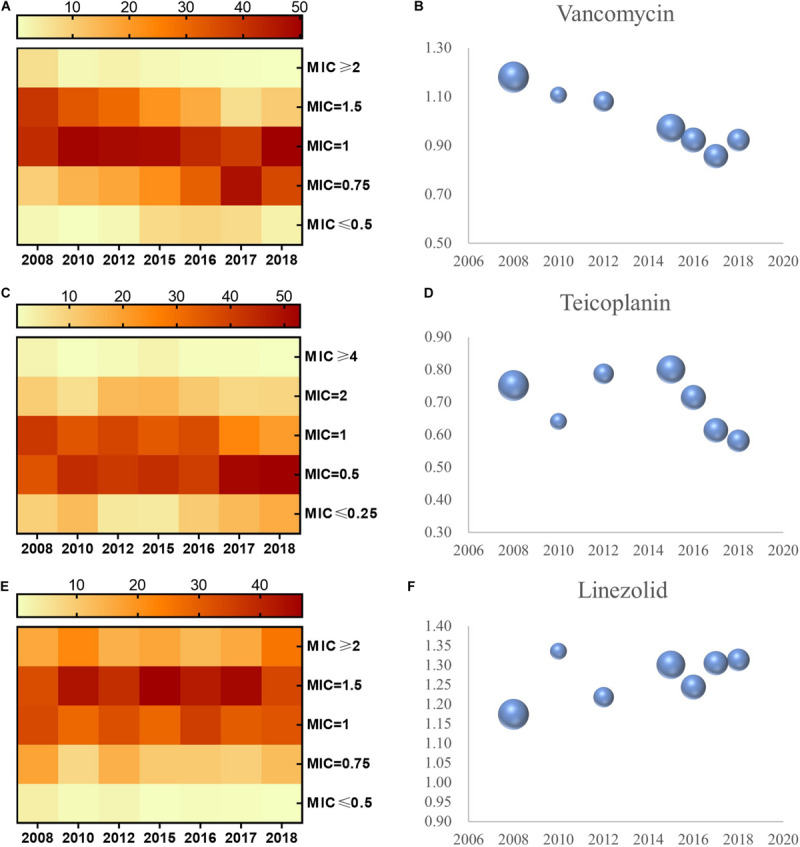
MIC distribution and the geometric mean of MIC values for vancomycin, teicoplanin, and linezolid in *S. aureus*, 2008–2018. MIC distribution of *S. aureus* to vancomycin, teicoplanin, and linezolid **(A,C,E)**. Geometric mean MIC determined by E-test method over the study period **(B,D,F)**. The bubble size represents sample size of isolates.

Distribution of MICs for vancomycin, teicoplanin, and linezolid in *S. aureus* is shown in the heat map (Figures 1A,C,E). The proportion of vancomycin MIC >1.0 mg/L was reduced from 2008 to 2018, while the proportion of vancomycin MIC <1.0 mg/L increased during this period. The proportion of MIC > 1.0 and <1.0 mg/L displayed the same alteration for teicoplanin in *S. aureus*. However, the distribution of the MIC for linezolid in *S. aureus* manifested differently, with an increase in the proportion of MIC > 1.0 mg/L versus a decrease in MIC < 1.0 mg/L. Linear regression chi-square test showed statistically significant differences (*p* < 0.001 for vancomycin, *p* < 0.001 for teicoplanin, and *p* < 0.001 for linezolid).

Apart from a distinct shift in the distribution of MIC for the aforementioned three antimicrobials, the concrete MIC value trended in the same way. We evaluated the geometric mean MIC for vancomycin, teicoplanin, linezolid, and isolate quantity from 2008 to 2018 (Figures 1B,D,F). In *S. aureus*, the phenomena of MIC decline for vancomycin and teicoplanin and MIC creep for linezolid can be observed in Figure 1. Non-parametric Spearman correlation test identified statistical significance (with correlation coefficient and *p-*value of -0.354 and <0.001 in vancomycin, -0.136 and *<*0.001 in teicoplanin, 0.07 and 0.001 in linezolid, respectively). Furthermore, when the MICs to these three antimicrobials in *S. aureus* were examined with paired-sample geometric *t*-test, we found a striking significance between the MIC for vancomycin and the MIC for teicoplanin (with correlation coefficient and *p-*value of 0.211 and *<*0.001), while no significance was observed in *S. aureus* between the MIC of linezolid and MIC of vancomycin (*p* = 0.114), and neither for the MIC for linezolid and MIC for teicoplanin (*p* = 0.194).

### Vancomycin MIC Decline Is Notably Exhibited in Both MRSA and MSSA

**FIGURE 2:**
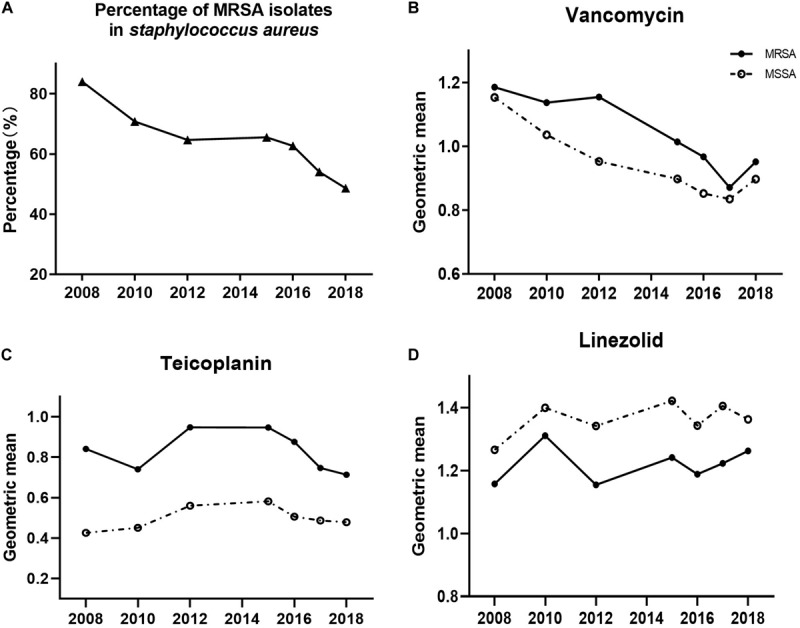
Shifts of MRSA proportion **(A)**, and MICs of **(B)** vancomycin, **(C)** teicoplanin, and **(D)** linezolid in MRSA (solid circles) or MSSA (open circles), 2008–2018.

A significant reduction was observed in the proportion of MRSA during the study period, ranging from 84% in 2008 to 49% in 2018 (Figure 2A, *p <* 0.001). When the shifts of the three antimicrobials’ MICs were analyzed with this reduction of MRSA proportion, we found that only vancomycin’s MIC decline was prominent relative to the declining proportion of MRSA (*p* = 0.006).

Additionally, we assessed the MIC value of the three antimicrobials in MRSA and MSSA to determine if they progressively shifted with annual trends (Figures 2B,C,D). We observed a notable significantly decreasing trend of the MIC for vancomycin in both MRSA and MSSA (both with *p* < 0.001). Likewise, teicoplanin’s MIC decline was identified in MRSA (*p* = 0.037), whereas linezolid in MRSA as well as teicoplanin and linezolid in MSSA exhibited no statistically distinct trends of MIC creep or decline.

Taking into consideration the MICs of MRSA and MSSA to the same antimicrobial, the MICs of vancomycin in MRSA isolates were higher than those in MSSA isolates (Figure 2B, *p* < 0.001). This trend of MRSA isolate MICs being higher than those of MSSA MICs was recapitulated for teicoplanin (Figure 2C, *p* < 0.001), whereas the inverse was observed for linezolid (Figure 2D, MSSA MIC higher than MRSA MIC, *p* < 0.001).

### Dominating MRSA Clone ST5 Exhibited an Analogous in Vancomycin’s MIC

**FIGURE 3:**
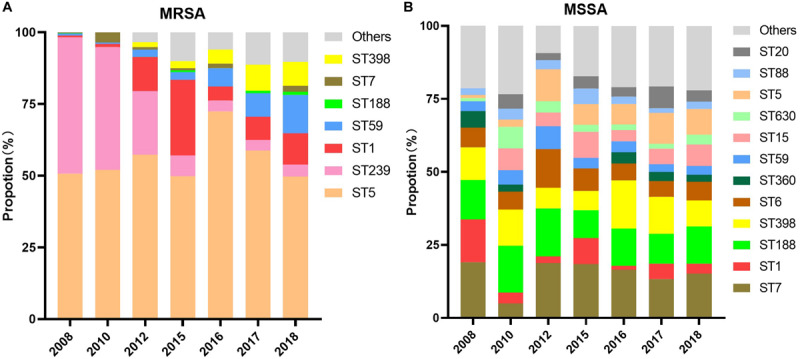
Composition of clones of **(A)** MRSA and **(B)** MSSA, 2008–2018.

The composition of MRSA and MSSA clones from the years 2008 to 2018 was analyzed and summarized in Figure 3. The clonal composition of MSSA was of higher diversity than MRSA. Although the ST5 clone dominated MRSA with more than 50% every year during the study period, it comprised a small part of MSSA clones with an increased proportion from 1% in 2008 to 9% in 2018; while the MRSA ST239 clone exhibited a dramatic decline from 48% in 2008 to 4% in 2018.

Minimum inhibitory concentration changes in the three aforementioned antimicrobials within different clones in MRSA or MSSA were examined via non-parametric Spearman correlation test during the study period. Among MRSA clones, vancomycin MIC decline was observed in ST5, ST239, ST398, and ST1 clones (*p* < 0.001, *p* = 0.040, *p* = 0.002, and *p* < 0.001, respectively), and linezolid MIC creep was observed solely in ST5 (*p* < 0.001). Surprisingly, both MRSA ST5 and ST239 clones exhibited teicoplanin MIC creep (*p* < 0.001 and *p* = 0.028, respectively), which was opposite to the teicoplanin MIC decline exhibited by overall MRSA. No statistical significance of trend in MIC changes to three antimicrobials in other clones was observed.

### Vancomycin MIC Decline Was Associated With an Increase in the Clinical Consumption of Linezolid

Vancomycin and linezolid DDDs (DDD/100 patients-day) from 2012 to 2018 (information not available before 2012) in this teaching hospital were collected in this study (Table 2). The significantly increasing trend of linezolid consumption in this period (*p* = 0.003) was notable, while no differences in vancomycin usage were observed (*p* = 0.702). Trends in antimicrobials usage and MIC changes during the period were calculated with the non-parametric Spearman correlation test. An overwhelming significance was found in the correlation between vancomycin MIC and linezolid consumption (with correlation coefficient and *p-*value of -0.975, 0.005). It is also worthy of mentioning that a positive relation existed between linezolid MIC change and linezolid consumption, although no significant variation was achieved (with correlation coefficient and *p-*value of 0.872, 0.054).

**TABLE 2:** Vancomycin and linezolid consumption (DDDs), 2012–2018.

**Year**	**2012**	**2013**	**2014**	**2015**	**2016**	**2017**	**2018**	***p-*value**
VAN	18.56	14.27	19.01	15.79	14.98	20.05	17.66	0.702
LNZ	6.53	7.12	6.86	7.8	10.76	14.5	12.97	0.003

*Data of vancomycin and linezolid daily defined doses (DDDs, an indicator of antimicrobials usage) in 2012–2018 in this teaching hospital were gathered and calculated. Thereinto, no differences in vancomycin usage were observed (*p* = 0.702), while increasing trend of linezolid consumption in this period (*p* = 0.003) was notable.*

## Discussion

Studies reporting vancomycin MIC creep in *S. aureus* have produced conflicting results, and even large multi-center surveillance studies displayed controversial results (Sader et al., 2009; Ho et al., 2010; Ruiz et al., 2016; Diaz et al., 2018). The sensitivity of the antimicrobial susceptibility testing method to detect MIC variations might account for these discrepancies to a certain extent, especially for vancomycin with a narrow therapeutic window. The broth microdilution (BMD) method and E-test method are both recommended for MIC testing according to the guidelines published by CLSI (CLSI, 2006). For many laboratories, automated systems are in routine use rather than E-test or BMD for assessing the antibiotic susceptibility nowadays indeed. Besides, researchers found inter-method variation in MICs obtained from the same isolates, irrespective of antibiotic, and MIC might change through storage of isolates (van Hal et al., 2011). However, compared with the BMD method, the E-test method is easier to operate as well as less prone to contamination. Using a gradient of antibiotic concentration, E-test method has greater precision and better ascertainment of the actual MIC than disc diffusion methods (Baker et al., 1991; Jorgensen and Ferraro, 2009). Antimicrobial susceptibility assessment of all of the 2911 sequential and non-repetitive *S. aureus* isolates collected in total for this study was carried out by the E-test method, which laid a solid foundation for the further statistical analysis of MIC change.

Moreover, the methods to evaluate MIC data, while defining “MIC creep,” were diverse in different research centers. A slight but statistically significant increase in percentage of MIC > 1.0 or 1.5 mg/L, or less-sensitive markers MIC50 and MIC90, as well as shifts in geometric mean MIC, could each be described as MIC creep (Steinkraus et al., 2007; Ho et al., 2010; Motoyasu et al., 2014; Hsieh et al., 2016). Besides, the calculation of the significance of vancomycin MIC creep was diverse among researchers. Actually, a portion of scholars referred growth in percentage of isolates with MIC more than 1.5/2.0 mg/L as vancomycin MIC creep (Delgado et al., 2007), as setting a lower clinical breakpoint might lead to a loss of reproducibility and frequent misclassification of susceptibility. However, as early in 2006, the CLSI lowered *S. aureus* vancomycin MIC breakpoint from 4 to 2 mg/L, as evidence showed that vancomycin has reduced efficacy against isolates with 4 mg/L (Tenover and Moellering, 2007). Increase in population of isolates with MIC > 1.0 mg/L was reported to define vancomycin MIC creep (Steinkraus et al., 2007; Ho et al., 2010; Zhuo et al., 2013), and vancomycin MIC> 1 mg/L were associated with higher treatment failure rates and mortality in patients with serious *S. aureus* infections like bacteremia, hence many studies chose 1.0 mg/L as clinical vancomycin breakpoint (Cervera et al., 2009; Gould, 2010). In this study, we evaluate variation in proportion of isolates with vancomycin MIC more than 1 mg/L and a reduction was found. Moreover, utilizing one traditional susceptibility marker individually as proof of MIC creep might hardly be convincing, as an increase in the frequency of isolates for which MICs are elevated does not indicate an increase in the central tendency of the MIC. In our study, the MIC data of each *S. aureus* isolate were systematically collected and analyzed with relevant years in manner of non-parametric Spearman correlation test. Simultaneously, geometric mean MIC, MIC50, MIC90, and MIC range were evaluated to ensure the inclusion of typical and representative results. At length, we demonstrated in *S. aureus* a decline in MIC for vancomycin and teicoplanin, and MIC creep for linezolid.

A dramatic reduction in the proportion of MRSA is now acknowledged nationwide in China, from 69% in 2008 to 35% in 2017 as announced by CHINET (Hu et al., 2018). This diminution in the proportion of MRSA was associated with a statistically significant vancomycin MIC decline in this study, offering convincing evidence that the observed vancomycin MIC decrease might be caused by a concomitant decline in the scale of MRSA infections. On the other hand, we found that vancomycin MIC decline was identified in both MRSA and MSSA, while a teicoplanin MIC decline was only evident in MRSA. MRSA showed a more characteristic trend in MIC change confronted with MSSA. For the treatment of MRSA, vancomycin was still the first choice, although isolates with high MIC value within the susceptible range resulted in higher mortality and worse prognosis (Li et al., 2017).

Vancomycin and teicoplanin are members of the glycopeptide family, which is a group of glycosylated cyclic or polycyclic non-ribosomal peptides that inhibit Gram-positive bacterial cell-wall synthesis (Zhuo et al., 2013; Zeng et al., 2016). Yet linezolid, a new class of oxazolidinones antibacterial agents, inhibits bacterial protein synthesis by blocking the formation of the 70S initiation complex (Birmingham and Schentag, 2003; Yue et al., 2016). Owing to the discrepancy between glycopeptide and oxazolidinone agents, vancomycin displayed a similar MIC profile, such as MIC decline of *S. aureus*, as teicoplanin in our research, in marked contrast to that exhibited by linezolid.

One of the most important findings of this study was the correlation between MIC changes and *S. aureus* clonotypes. In our recent work, MRSA exhibited a dramatic decline in the prevalence of the ST239 clone, from 41% in 2008 to 2% in 2017, while ST5, whose proportion did not significantly change during the same time frame, represented the major clone among MRSA isolates (Dai et al., 2019). Taking into account the respective primary clone types, in the present study, we observed a strikingly identical trend in terms of the dynamic changes to the MIC of the critical antimicrobial vancomycin in overall MRSA isolates. The ST5 clone constituted the overwhelming majority clone type in MRSA, in which vancomycin’s MIC declined in a statistically obvious manner, recapitulating the same tendency of MIC decline in overall MRSA. Even in overall ST239 clones, despite the dramatic decline in prevalence, we identified a similar trend of vancomycin MIC decline. We can thus draw the conclusion that the observed MIC dynamic changes did not result from shifts in the molecular epidemiology of *S. aureus*, as the dominating ST5 and pre-dominating ST239 clones exhibited the same trend as that of MRSA against vancomycin. However, higher diversity of clonotype composition in MSSA compared to MRSA resulted in no statistical significance of a trend in MIC changes to the three antimicrobials in the majority of clones.

“Regulations for Clinical Application of Antibacterial Agents,” issued by the Ministry of Health of the PRC in 2012, provided a legal guarantee for the rational use of antibacterial agents in China and, as a result of which, clinical medication achieved a standardization, pledging antimicrobials resistance steerable (Xiao, 2012). Since reports had identified a relationship between MIC change and antimicrobial consumption (White et al., 2000; Pol and Ruegg, 2007; Hansen, 2010), to further elucidate the relationship between antimicrobial consumption and their respective MICs in *S. aureus* in this teaching hospital, we gathered DDDs of vancomycin and linezolid from 2012 to 2018. We observed that linezolid consumption increased dramatically, and that patterns of antimicrobial usage varied as indications of linezolid and teicoplanin were broadened on the basis of updated guidelines (Hassoun et al., 2017; Li et al., 2017) on account of which vancomycin MIC decline and linezolid MIC creep were identified in *S. aureus* during the study period of 2008–2018. However, further enhancement in the database construction of dynamic changes of MICs for vital antimicrobials is still indispensable; therefore, it might provide an epidemiological basis for corresponding measures for the rational use of antimicrobials.

This research has some limitations. First, some of the isolates from 2010 and 2012 could not be revived, and isolates from 2009, 2011, 2013, and 2014 were not included. Second, MIC decline of vancomycin and teicoplanin, and MIC creep of linezolid were statistically significant in *S. aureus*, while the difference was somewhat slight. Third, MIC decline exhibited in vancomycin was found to be associated with the increase of linezolid consumption according to the statistical analysis; however, direct evidence or possible molecular mechanisms was not provided. In the following study, whole-genome sequencing (WGS) of a certain number of representative *S. aureus* isolates, followed by co-analyzing with their clonotypes and changes of susceptibility to vancomycin, linezolid, and teicoplanin will be carried out.

To sum up, via analysis in dynamic changes of *S. aureus* susceptibility to antimicrobials, MIC decline of vancomycin was identified both in MRSA and MSSA, and both the dominating MRSA clone ST5 and pre-dominating MRSA clone ST239 displayed vancomycin MIC decline, while teicoplanin MIC decline was only identified in MRSA. Linezolid MIC creep was identified in total *S. aureus*, but linezolid in MRSA as well as teicoplanin and linezolid in MSSA displayed no statistically distinct trends of MIC creep or decline. Clinical consumption of linezolid was correlated with vancomycin MIC decline in *S. aureus*, suggesting that changes in clinical antibiotic use may affect bacterial resistance.

## Data Availability Statement

All datasets generated for this study are included in the article/supplementary material.

## Ethics Statement

The bacteria from patient samples were approved by the ethics committee of Renji Hospital, School of Medicine, Shanghai Jiao Tong University, Shanghai, China. This project is a retrospective study. All of the *S. aureus* isolates were cultured and identified in routine microbiology laboratories. It did not involve the collection of patients’ clinical information, and did not interfere with patients’ clinical treatment. Patients were not involved in any way in the study, only molecular analysis of the bacteria was performed, thus, informed consent was not required for participation in this study.

## Author Contributions

YJ, HL, and ML contributed to conception and design of the study. HL, JL, and YL organized the database. YJ performed the statistical analysis. YJ and HL plotted the figures and tables in this work. YJ and HL wrote the first draft of the manuscript. JL, YL, QH, QL, and ML wrote sections of the manuscript. All authors contributed to manuscript revision and read and approved the submitted version.

## Conflict of Interest

The authors declare that the research was conducted in the absence of any commercial or financial relationships that could be construed as a potential conflict of interest.

## Footnotes

1 http://www.pubmlst.net
